# Don't Make Me Angry: Frustration-Induced Anger and Its Link to Aggression in Women With Borderline Personality Disorder

**DOI:** 10.3389/fpsyt.2021.695062

**Published:** 2021-05-28

**Authors:** Katja Bertsch, Sarah Back, Aleya Flechsenhar, Corinne Neukel, Marlene Krauch, Karen Spieß, Angelika Panizza, Sabine C. Herpertz

**Affiliations:** ^1^Department of Psychology, Ludwig-Maximilians-University Munich, Munich, Germany; ^2^Department of General Psychiatry, Medical Faculty, Center for Psychosocial Medicine, Heidelberg University, Heidelberg, Germany; ^3^Department of Psychosomatic Medicine and Psychotherapy, Central Institute of Mental Health, Medical Faculty Mannheim, Heidelberg University, Heidelberg, Germany

**Keywords:** negative affect, emotion regulation, anger, reactive aggression, interpersonal dysfunction

## Abstract

Aggression is a prominent interpersonal dysfunction of individuals with borderline personality disorder (BPD). In BPD aggression is predominantly reactive in nature, often triggered by frustration, provocation, or social threat and is associated with intense anger and an inability to regulate this strong, negative emotion. Building on previous research, we were interested in investigating negative emotionality in general and anger in particular in women with BPD before and after frustration induction. To achieve this, 60 medication-free women with BPD and 32 healthy women rated the intensity of negative emotions (angry, frustrated, upset, embarrassed, nervous) before and after performing a Titrated Mirror Tracing Task, which reliably induces frustration and distress. As expected, women with BPD reported significantly greater intensity of negative emotions before and after frustration than healthy women. Specifically, they showed a significantly stronger frustration-induced increase in anger, while other negative emotions remained unaffected by frustration induction. This anger increase was significantly related to aggressive behavior reported in the 2 weeks prior to the experiment, as well as to the level of frustration experienced in the experiment itself, but not with emotion dysregulation. The current data confirm the important role of frustration-induced anger independent of emotion dysregulation in BPD, in particular with regard to aggression, a prominent interpersonal dysfunction of this disorder. These findings underline the importance of interventions with particular focus on anger.

## Introduction

Borderline personality disorder (BPD) is characterized by severe and lasting impairments in self- and interpersonal functions with emotion dysregulation as core symptom (1). Emotion dysregulation has been conceptualized in BPD as increased sensitivity, high intensity and labile negative emotional states, deficits in appropriate regulation strategies, and a surplus of maladaptive regulation strategies (2). One of these maladaptive regulation strategies might be aggression (3). Aggression can be defined as any behavior that intends to harm another individual against their will (4). With over 70% of patients acting out aggressively against others within a year, aggression is frequent in BPD (5). Being predominantly reactive in BPD, aggression is triggered by frustration, provocation, or threat, closely related to feelings of anger, and causes severe interpersonal problems (3).

Several cross-sectional and longitudinal studies have shown emotion dysregulation to be an important mediator of aggression in BPD (6–8). Individuals with BPD report more negative emotions and a greater intensity of negative emotions than healthy individuals throughout the day (9). However, recent data suggest a particular relevance of anger, a negative emotion that is closely related to reactive aggression, in BPD. Using e-diaries, Kockler et al. (10) found that individuals with BPD exhibit anger more frequently in their daily life than healthy as well as clinical control groups and feelings of anger accounted for more distress than pure emotional intensity. In several other studies more intense anger experiences, higher proneness to anger, prolonged anger reactions, as well as higher anger rumination have been reported in individuals with BPD (11–14) and these intense feelings of anger seem to persist across the lifespan (15). Furthermore, individuals with BPD seem to perceive neutral or ambivalent faces more often as angry (“anger bias”) in experimental face perception studies than healthy controls [for reviews, see (16, 17)]. In healthy individuals, frustrating or provoking (experimental) situations increase feelings of anger, thereby increasing the likelihood of aggressive reactions (18). Using cross-sectional self-report data, a similar sequalae appeared in BPD where emotion dysregulation and trait anger sequentially mediated the association between BPD symptomatology and aggression (6).

Taken together, these studies suggest a particular relevance of anger in BPD – particularly with regard to aggression. We therefore aim to investigate whether individuals with BPD show greater intensity of negative emotions in general and of anger in particular, at baseline as well as in response to frustration. Furthermore, we were interested in researching whether frustration-induced anger would be stronger in those individuals with BPD who tend to act out aggressively in their everyday life and have difficulties regulating their emotions.

To achieve this, a relatively large medication-free sample of women with BPD and a matched healthy controlled group took part in a computerized version of the Titrated Mirror Tracing Task [TMTT; (19)], which reliably induces frustration and distress [e.g., (20, 21)], in which they were asked to rate their current negative emotional state (including anger) before and after the frustration induction. Importantly, the frequency and intensity of aggressive behavior in the 2 weeks before the experiment was assessed by trained interviewers with the modified Overt Aggression Scale [OAS-M; (22)]. We hypothesized that women with BPD would report greater intensity of negative emotions in general than healthy women before and after frustration (hypothesis 1). We further expected the frustration-induced anger increase in particular, to be significantly stronger in women with BPD than in healthy women (hypothesis 2). Finally, we thought that a frustration-induced increase of anger would be higher in those women with BPD who act out aggressively more often in their everyday life and show higher deficits in emotion regulation (hypothesis 3). Additionally, we examined the effects of anger intensity before the experiment as well as experienced frustration within the experiment on frustration-induced increase in anger.

## Methods and Materials

### Participants

The sample consisted of *N* = 60 women with a current DSM-IV diagnosis of BPD (BPD; mean age = 29.6 years SD = 7.9, range: 19–49 years) and *N* = 32 healthy women (HC; mean age = 28.7 years SD = 8.8, range: 18–50 years). Groups were matched in terms of age [*t*_(90)_ = 0.48, *p* = 0.634] and intelligence [*t*_(91)_ = −0.68, *p* = 0.500, see Table 1]. Based on a priori power analyses, a sample of *N* = 60 women with BPD and *N* = 30 HC was intended in order to detect small group by emotion interactions in a repeated measures analysis of variance (*f* ^2^ = 0.02) at a significance level of α = 0.05 with a statistical power of 1-ß > 0.94. The sample of *N* = 60 women with BPD is also large enough to detect medium to large effects (*R*^2^ = 0.20) in a linear multiple regression with up to five predictors at a significance level of α = 0.05 and a statistical power of 1-ß = 0.82.

**Table 1:** Sociodemographic and self-report data of women with borderline personality disorder (BPD; *N* = 60) and healthy controls (CON; *N* = 32).

	**BPD (*M* ±*SD*)**	**CON (*M* ±*SD*)**	***t***	***p* (uncorrected)**
Age (in years)	29.6 ± 7.9	28.7 ± 8.8	0.48	0.634
Intelligence (Raven)	112 ± 10.6	113.5 ± 10.6	−0.68	0.500
ADHD-symptoms (ADHS-SB)	22.0 ± 10.0	2.6 ± 3.4	9.76	<0.001
Aggression (OAS-M)	14.8 ± 12.4	0.6 ± 1.6	8.82	<0.001
**Emotion dysregulation (DERS)**				
- Total score	131.7 ± 19.9	64.8 ± 16.3	16.34	<0.001
- Non-acceptance	22.0 ± 6.2	11.0 ± 5.6	8.43	<0.001
- Goals	21.7 ± 3.3	11.2 ± 4.5	11.82	<0.001
- Impulse	19.8 ± 5.6	8.8 ± 3.1	12.32	<0.001
- Aware	19.9 ± 5.2	14.3 ± 3.7	5.38	<0.001
- Strategies	29.8 ± 6.6	11.5 ± 3.7	17.07	<0.001
- Clarity	18.2 ± 4.0	8.0 ± 3.3	12.34	<0.001
**Comorbidities**				
affective disorders	27 (48)			
anxiety disorders	43 (54)			
posttraumatic stress disorder	15 (22)			
eating disorders	17 (36)			
somatic syndrom disorders	5 (0)			
avoidant personality disorder	18 (19)			
antisocial personality disorder	1 (2)			

*Comorbid disorders [N_current_ and (N_lifetime_)] of women with BPD; please note that life time mental disorders were exclusion criteria for CON. M, mean; SD, standard deviation; Raven; ADHD-SB; OAS-M; DERS*.

General exclusion criteria comprised neurological disorders, severe medical illness, or intake of psychotropic medication for at least 2 weeks prior to participation. Further exclusion criteria for the BPD group were alcohol/drug abuse in the last 2 months or alcohol/drug dependency in the last 12 months (as assessed by urine drug screening and interview), as well as lifetime diagnosis of schizophrenia, schizoaffective, or bipolar disorder. Healthy controls were excluded if they had any lifetime DSM-IV diagnosis and/or current or past psychotherapeutic or psychiatric treatment. Details on sample characteristics including comorbidities are provided in Table 1.

The study was part of a larger project on social information processing within the German research consortium KFO-256 (pre-registration at www.drks.de: DRKS00009551). Participants were recruited through a KFO-256 recruitment unit with psychometric data of all participants monitored in a central database [for details about the KFO-256, see (23)]. Individuals with BPD were recruited from all over Germany (including inpatients and outpatients of specialized units for personality disorders, referrals from practitioners as well as social media and newspaper advertisements). Samples across KFO-256 studies may show overlap in participants. The study was approved by the Ethics Committee of the Medical Faculty of the University of Heidelberg, Germany. Participants provided written informed consent and were financially reimbursed.

### Experimental Protocol

All participants took part in a diagnostic process including an extensive telephone screening as well as an onsite diagnostic appointment, where all interviews were conducted face-to-face by trained diagnosticians. Questionnaires were delivered as paper-and-pencil or computerized versions to each participant individually. At the day of the experiment, they performed a urine toxicology screening for exclusion of acute substance abuse. Participants also took part at three further computer tasks on the same day, which did not include frustrations or otherwise arousing conditions. The sequence of tasks was randomized across participants and experimenters were instructed to keep breaks between tasks.

### Diagnostic and Self-Report Measures

*BPD diagnosis* and *comorbid disorders* were determined with the Structured Clinical Interview for DSM-IV Axis I Disorders [SCID-I; (24)] and the International Personality Disorder Examination [IPDE; (25)]. All interviews were performed by experienced diagnosticians with a M.Sc. in Psychology and standardized training (inter-rater reliability: ICCs ≥ 0.911).

*Aggressive behavior in the 2 weeks before the experiment* was assessed with the *Modified Overt Aggression Scale* [OAS-M; (22)]. This semi-structured interview captures the frequency and severity of overt aggressive behavior including verbal assault, assault against objects, others, and self as well as irritability. The sum of all subscales assessing aggressive behaviors (i.e., verbal assaults, assaults against objects and others) was used. High interrater reliability (ICCs = 0.96–0.98) has been documented for this instrument (26).

*Emotion dysregulation* was assessed with the Difficulties in Emotion Regulation Scale [DERS; (27)]. This self-report questionnaire measures typical levels of emotion dysregulation across six domains: non-acceptance of negative emotions (subscale: non-accept), inability to engage in goal-directed behaviors when experiencing negative emotions (subscale: goals), difficulties controlling impulsive behaviors when experiencing negative emotions (subscale: impulse), limited access to emotion regulation strategies perceived as effective (subscale: strategies), lack of emotional awareness (subscale: awareness), and lack of emotional clarity (subscale: clarity). The DERS contains 36 items that are rated on a 5-point scale (from 1 = almost never to 5 = almost always) and are summed to create a measure of emotion dysregulation (total score). The possible scores can range from 36 to 180, with higher scores indicating greater emotion dysregulation. Internal consistency in the sample under study was found to be high (Cronbach's α = 0.97 for the total scale and 0.81–0.95 for the subscales).

To control for possible confounding effects of *intelligence* or *Attention Deficit and Hyperactivity Disorder (ADHD) symptoms*, the following two measures were used. Since only one of the patients had a DSM-IV diagnosis of *Antisocial Personality Disorder* (ASPD), this was not added as a further control variable.

*Intelligence* was estimated with the Raven's Standard Progressive Matrices [SPM; German Version; (28)]. This test entails 60 items that ask the participants to identify the correct missing piece to complete a larger pattern. Possible scores can range from 0 to 60, with higher scores indicating higher IQ. Raw values were transformed to IQ-scores. Across studies, Raven's standard progressive matrices were shown to have acceptable to excellent internal consistency [Cronbach's α = 0.77–0.96; (29)].

*ADHD-symptoms* were assessed with the *ADHD self-rating scale* [ADHS-SB; (30)], a 22-item questionnaire with a 4-point scale (0 = strongly disagree to 3 = strongly agree) that can be summed up to a total score. The possible scores can range from 0 to 66, with higher scores indicating greater likelihood of symptoms of ADHD. Internal consistency for the ADHD self-rating scales in the current sample was found to be excellent (Cronbach's α = 0.94).

### Titrating Mirror Tracing Task

The TMTT (19) was used to induce frustration and measure frustration-related anger. In this task, participants were required to trace a red dot along the lines of a star using the computer mouse. However, the mouse was programmed to move the red dot in the reverse direction, that is, if the participant moved the mouse to the left, the red dot would move to the right etc. To further increase the difficulty of this task and the resulting frustration, moving the red dot outside of the lines of the star or stalling for more than 2 s caused a loud buzzing noise and the red dot to return to the starting position.

The task consisted of four rounds in total: three rounds with increasing difficulty to induce frustration and one final round that could be self-terminated to measure frustration tolerance. In the first three rounds, difficulty was increased by titrating the star width based on the participant's individual performance in the prior star. All participants started with a first, *easy star* (line width: 30 pixel and total distance: 1,470 pixels, duration: 2 min). After finishing, the participants were informed that the next star would be more difficult. The line width of this second, *medium star* was determined by dividing the participant's best distance by 98 pixels (1,470 pixels/15) rounding down and then subtracting this number from 30 which resulted in a line width between 15 and 30 pixels (duration: 2 min). After this, participants had to work on a third, *hard star* for 1 min. This star's line width was 5 pixels smaller than the line width of the medium star. The fourth and *final star* had the same line width as the hard star, but this time, participants were informed that they could end the task at any time by pressing the space bar. They were not informed of the maximum duration of 5 min prior to beginning this final round. The latency in seconds to task termination in this final round has been found a reliable measure for frustration tolerance [e.g., (20, 21)]. To check for group differences in task performance and difficulty levels, line widths, total number of errors, and percentage of the star the participants covered on their best attempts for each star were analyzed.

The intensity of negative emotions was assessed before and after frustration induction with the Positive and Negative Affect Scale [PANAS; (31)]. Unlike the original PANAS' 0–10 scale, participants were asked to indicate their current emotional state (e.g., anger) on a continuous scale from 0 (none) to 150 (extreme). Each emotional state was presented on a separate screen. As in the original PANAS, the following negative emotional states were assessed: angry, frustrated, upset, embarrassed, nervous.

The TMTT significantly correlates with other frustration tasks such as the Paced Auditory Serial Addition Task (PASAT) among different samples including individuals with BPD [e.g., (20, 21)].

### Statistical Analyses

All analyses were performed in IBM SPSS (version 24). For all statistical analyses, a significance threshold of *p* < 0.05, two-tailed, was set. Across all participants, 1.9% data were missing at random according to Little's MCAR-test [$\chi_{\left(241\right)}^{2}$ = 223.17, *p* = 0.789). Multiple imputation was conducted in case of missing values.

Prior to the analyses of interest, normal distribution for all variables of interest was explored by examining skewness and kurtosis indices and normality plots (Q-Q-plots). Normal distribution was regarded as adequate for all data. Linearity could be confirmed by visual inspection of bivariate scatterplots and homoscedasticity and independence of error terms with univariate scatterplots. Furthermore, Durbin-Watson statistic was acceptable [*d* = 2.17; (32)]. Multicollinearity was checked with the variance inflation factor (VIF), which reached a maximum of 1.41, indicating that multicollinearity did not appear to be a problem in the current study (32).

Group differences in socio-demographic data, self-reports, frustration tolerance, and performance in the TMTT were assessed with independent sample *t*-tests. Effect sizes are reported as Cohen's *d* (33).

For our first *research question*, namely, whether women with BPD would show greater intensity of negative emotions before and after frustration than healthy women, we performed a group by emotion by time mixed-design analysis of variance (ANOVA) to test for group differences in the five negative emotional states before and after the frustration induction. Independent and paired *t*-tests with Bonferroni correction for multiple testing were used as *post-hoc* tests. Effect sizes of significant results are reported as proportion of explained variances (η^2^). Where appropriate, we applied the Huynh-Feldt procedure (34) to correct for potential violations of the sphericity assumption.

Our second *research question*, i.e., whether women with BPD would show a particular increase in anger after frustration, we submitted the differences scores (post- minus pre-frustration emotional intensity ratings) to independent sample *t*-tests using Bonferroni correction for multiple testing and reporting Cohen's *d* as effect sizes.

For our *third and final research question*, i.e., whether frustration-induced anger increase was related to aggressive behavior and emotion dysregulation in BPD, we ran a series of hierarchical linear regressions. In the first block, we entered the relevant covariates (age, intelligence, and ADHD symptoms). In block 2, aggressive behavior in the past 2 weeks and self-reported dysregulation of negative emotions were entered (see **Table 3**). In a third block, we additionally entered the intensity of anger before frustration to examine whether frustration-induced anger increase was stronger in those individuals with higher levels of anger at baseline. Finally, in the fourth block, the total number of errors at the hard star was entered to investigate whether frustration-induced anger increase, beyond effects of baseline anger levels, depended on the individual frustration level. Note that this measure reflects the number of loud buzzing noises along with returns to the starting position in the most difficult condition.

Since the DERS subscale goals, which captures “the ability to engage in goal-directed behavior and refrain from impulsive behavior, when experiencing negative emotions” [(27), p. 43] very well represents the emotion regulation capacities required to perform the TMTT, we decided to include this subscale rather than the total score in the regression analyses. Please note that hierarchical regression analyses were not conducted for the healthy control group because of limited sample size of *N* = 32 healthy women.

## Results

### Do Women With BPD Show a Greater Intensity in Negative Emotion Than Healthy Women Before and After Frustration?

**Figure 1:**
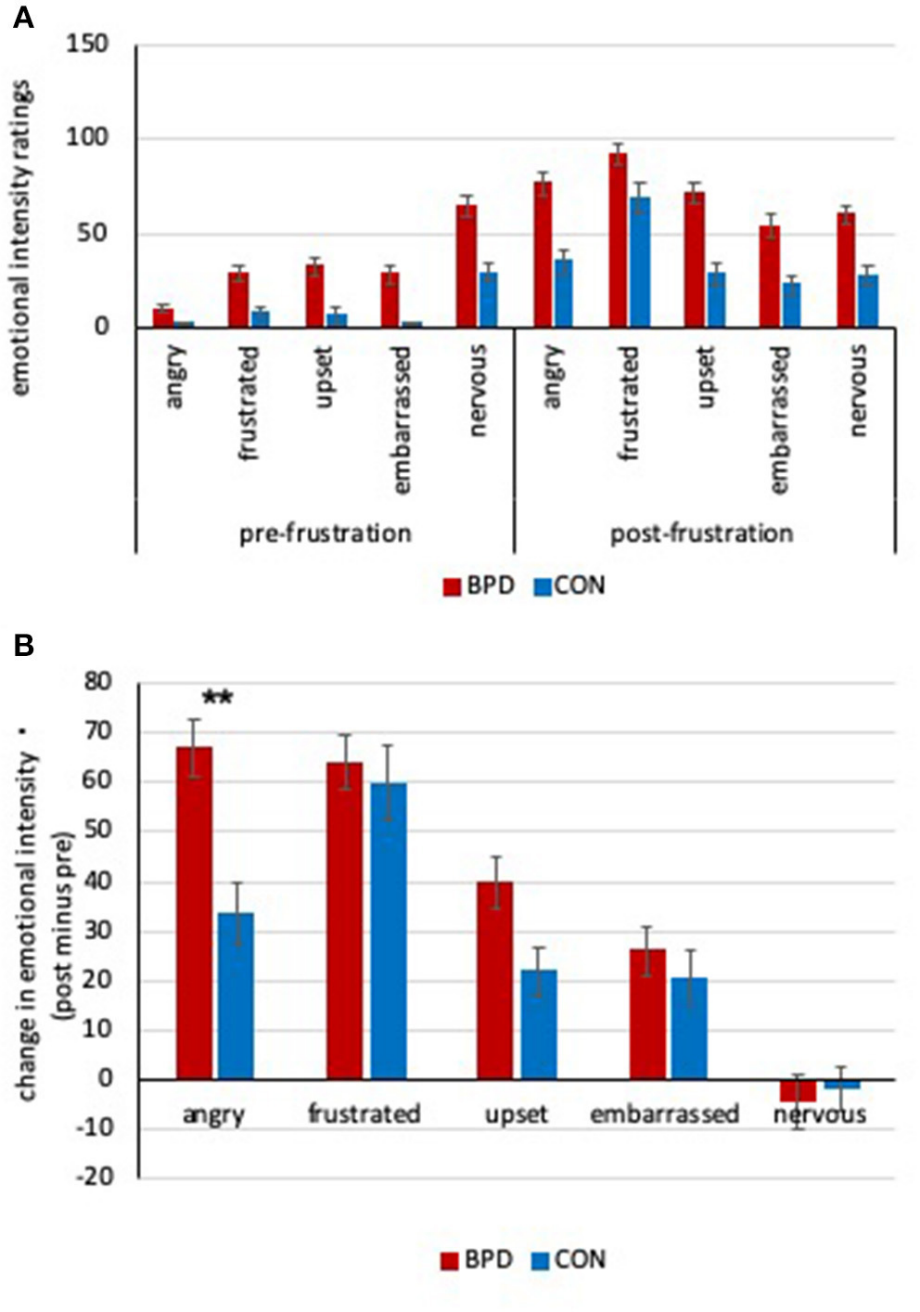
**(A)** Negative emotion intensity ratings before and after frustration from 0 to 150 of women with borderline personality disorder (BPD) and healthy controls (CON). See text for significances. **(B)** Frustration-induced changes in negative emotion intensity of women with BPD and healthy controls. Women with BPD showed significantly higher increase in anger (**p_Bonferroni_ < 0.001). Bars reflect group means and error bars indicate one standard error of the mean.

Women with BPD reported a significantly greater intensity of negative emotions than healthy women before and after frustration [main effect group, *F*_(1,90)_ = 28.10, *p* < 0.001, η^2^ = 0.24]. However, this main effect was qualified by significant group by time point [*F*_(1,90)_ = 4.01, *p* = 0.048, η^2^ = 0.04] and significant group by time point by emotion [*F*_(1,278.0)_ = 4.35, *p* = 0.005, η^2^ = 0.05] interactions. According to *post-hoc* tests, women with BPD were significantly angrier, more upset, embarrassed, and nervous than healthy controls before and after frustration [all *t*_(88.9)_ ≥ 3.74, *p*_Bonferroni_ < 0.001, *d* ≥ 0.76]. Women with BPD were also significantly more frustrated than healthy women before [*t*_(84.3)_ = 4.20, *p*_Bonferroni_ < .001, *d* = 0.81], but not after [*t*_(90)_ = 2.34, *p*_Bonferroni_ = 0.110, *d* = 0.51] the TMTT. Furthermore, women with BPD as well as healthy women were significantly angrier, more frustrated, upset, and embarrassed (but not more nervous) after than before frustration (see Table 2 and Figure 1A).

**Table 2:** Task performance and level of frustration at all four stars (easy, medium, hard, and last star).

	**BPD (*M* ±*SD*)**	**CON (*M* ±*SD*)**	***t***	***p* (uncorrected)**
**Easy star**				
Total no. of errors	30.8 ± 26.8	42.8 ± 39.7	1.54	0.132
% covered in best attempt	71.8 ± 29.3	62.5 ± 32.6	1.39	0.169
**Medium star**				
Line width	19.6 ± 4.6	21.0 ± 5.1	−1.42	0.160
Total no. of errors	29.8 ± 21.3	41.3 ± 36.1	−1.66	0.105
% covered in best attempt	46.6 ± 24.2	38.4 ± 20.7	1.62	0.109
**Hard star**				
Line width	14.6 ± 4.6	16.0 ± 5.1	−1.42	0.160
Total no. of errors	18.8 ± 13.2	21.7 ± 17.7	−0.88	0.381
% covered in best attempt	26.7 ± 18.3	22.7 ±12.2	1.10	0.275
**Last star**				
Total time spent on star	237.2 ± 141.3	249.9 ± 152.4	−0.40	0.690
Total no. of errors	57.7 ± 53.2	61.3 ± 39.8	−0.33	0.739
% covered in best attempt	49.2 ± 28.6	46.1 ± 33.2	0.46	0.648
**Pre-frustration negative emotion intensity**				
Angry	9.8 ± 14.0	2.0 ± 2.9	4.16	<0.001^*^
Frustrated	28.3 ± 32.4	8.4 ± 12.6	4.19	<0.001^*^
Upset	32.3 ± 39.0	6.3 ± 10.5	4.85	<0.001^*^
Embarrassed	28.2 ± 36.6	2.0 ± 2.0	5.53	<0.001^*^
Nervous	64.2 ± 41.2	29.1 ± 29.7	4.69	<0.001^*^
**Post-frustration negative emotion intensity**				
Angry	76.8 ± 47.0	35.3 ± 35.2	4.77	<0.001^*^
Frustrated	92.4 ± 47.0	68.3 ± 47.2	2.34	0.022
Upset	72.0 ± 43.9	28.0 ± 31.7	5.52	<0.001^*^
Embarrassed	54.2 ± 50.8	22.6 ± 30.1	3.74	<0.001^*^
Nervous	59.9 ± 38.5	27.4 ± 27.6	4.66	<0.001^*^
**Difference in emotion intensity (post minus pre)**				
Angry	67.0 ± 44.0	33.3 ± 35.0	3.74	<0.001^*^
Frustrated	64.0 ± 43.2	59.9 ± 43.2	0.44	0.661
Upset	39.7 ± 39.2	21.8 ± 28.3	2.52	0.014
Embarrassed	26.1 ± 39.0	20.6 ± 30.0	0.75	0.458
Nervous	−4.3 ± 42.1	−1.7 ± 25.5	−0.37	0.714

*Note that line width was always 30 pixels for the easy star, line width titration for all other stars depended on the participant's prior performance (line widths of hard and last stars are identical). Total number or error indicates the individual frustration level, since it reflects the number of loud buzzing noises and of returns to the starting position. The % covered in the best attempt reflects the participant's task performance. The total time spent on the last star indicates frustration tolerance. Pre-frustration negative emotion intensity ratings are provided before the easy star and post-frustration ratings after the hard star on a scale from 0 to 150. M, mean; SD, standard deviation;*

* *p = significant after Bonferroni correction for multiple testing*.

The ANOVA also revealed significant main effects of time point [*F*_(1,90)_ = 124.20, *p* < 0.001, η^2^ = 0.58] and emotion [*F*_(4,323.9)_ = 19.11, *p* < 0.001, η^2^ = 0.18], as well as a significant time point by emotion interaction [*F*_(4,278.0)_ = 53.3, *p* < 0.001, η^2^ = 0.37] which support the induction of distress in general and particularly of anger and frustration by the TMTT.

Hence, the current data support the previously reported greater intensity of negative emotions in women with BPD compared to healthy women at baseline, as well as after experiencing frustration.

### Does Frustration Particularly Increase Anger in Women With BPD Compared to Healthy Women?

Independent *t*-tests revealed that women with BPD showed a significantly stronger increase in frustration-induced anger than healthy women [*t*_(90)_ = 3.74, *p*_Bonferroni_ < 0.001, *d* = 0.85], while no significant group differences could be found for the four other negative emotional states [all *t*_(81.9)_ ≤ 2.52, *p*_Bonferroni_ ≥ 0.07, *d* ≤ 0.52, see Table 2 and Figure 1B].

Thus, although women with BPD generally reported a greater intensity of negative emotions in general, they specifically showed a higher frustration-induced increase in anger than healthy women.

### Is Frustration-Induced Anger Related to Aggressive Behavior and Emotion Dysregulation in Women With BPD?

Women with BPD reported significantly more aggressive behaviors in the 2 weeks before the experiment than healthy women [*t*_(62.7)_ = 8.72, *p* < 0.001 *d* = 1.61]. They also reported higher levels of emotion dysregulation in all six domains assessed by the DERS [total score: *t*_(90)_ = 16.36, *p* < 0.001, *d* = 3.68; six subscales: *t*_(90)_ ≥ 5.38, *p* < 0.001, *d* ≥ 1.24; see Table 2]. However, women with BPD neither showed differences in tolerance by quitting the very last star earlier [total time spent on last star: *t*_(90)_ = −0.40, *p* = 0.690, *d* = 0.09], nor performance [total number of errors: *t*_(90)_ = −0.33, *p* = 0.739, *d* = 0.08; percentage of last star managed by the participant: *t*_(90)_ = 0.46, *p* = 0.648, *d* = 0.10] than healthy women. Groups also did not significantly differ in their performance in the easy, medium, and hard stars [*t*_(90)_ ≤ 1.66, *p* ≥ 0.105, *d* ≤ 0.39, see Table 2 for details].

We then conducted a series of linear regressions that controlled for possible confounding variables to assess whether anger increase was related to aggressive behavior and emotion dysregulation in BPD. Our first model which only included the covariates age, intelligence, and ADHD symptoms was statistically not significant [*F*_(3,56)_ = 1.92, *p* = 0.137, *R*^2^ = 0.09; ß weights and changes in *R*^2^ are presented in Table 3]. In block 2, we entered aggressive behavior and emotion dysregulation. The omnibus regression model was significant [*F*_(5,54)_ = 3.20, *p* = 0.013, *R*^2^ = 0.23]. The effect of aggressive behavior on anger increase was significant (ß = 0.326, *t* = 2.70, *p* = 0.009), while the effect of emotion dysregulation was statistically not significant (ß = −0.216, *t* = −1.57, *p* = 0.122). In addition, intelligence emerged as a significant predictor (ß = −0.291, *t* = −2.22, *p* = 0.031). In block 3, we entered baseline anger, which failed to reach significance and did not add significantly to the model (see Table 3). Finally, in the fourth block, we entered total number of errors at the hard star as an indicator for the level of frustration. This effect was significant (ß = 0.297, *t* = 2.56, *p* = 0.014), and significantly improved the overall model fit [*F*_(7,52)_ = 3.43, *p* = 0.004, *R*^2^ = 0.32] as well. This final model accounted for 32% of variance in anger increase, thus indicating that increase in frustration-related anger is strongly and significantly related to aggressive behavior and experienced frustration, while controlling for possible confounding variables.

**Table 3:** The relationship of aggressive behavior, emotion dysregulation, baseline anger (pre-anger), and level of frustration (no. of errors at hard star) to anger increase.

**Predictor**	**ß**	**Full-model *R*^**2**^**	**Δ*R*^**2**^**	**Δ*F***
**Model 1**				
Age	0.028			
Intelligence	−0.312^*^			
ADHD-symptoms	−0.134	0.09	0.09	1.92
**Model 2**				
Age	0.047			
Intelligence	−0.291^*^			
ADHD-symptoms	−0.054			
Aggressive behavior	0.326^*^			
Emotion dysregulation	−0.216	0.23^*^	0.14	4.72^*^
**Model 3**				
Age	0.054			
Intelligence	−0.292^*^			
ADHD-symptoms	−0.051			
Aggressive behavior	0.314^*^			
Emotion dysregulation	−0.225			
Pre-anger	0.040	0.23^*^	0.001	0.09
**Model 4**				
Age	0.059			
Intelligence	−0.287^*^			
ADHD-symptoms	−0.077			
Aggressive behavior	0.305^*^			
Emotion dysregulation	−0.180			
Pre-anger	0.029			
Total no. of errors (hard star)	0.297^*^	0.32^*^	0.09	6.54^*^

* *p < 0.05*.

## Discussion

The current study confirmed a greater intensity of negative emotions in women with BPD compared to healthy women, at baseline, as well as after frustration induction. Furthermore, the frustration-induced anger in BPD, which was significantly higher than in healthy controls, was significantly related to aggressive behavior in the previous 2 weeks and the experienced frustration in the experiment.

The results are in line with those of previous ecological momentary assessments, which revealed more frequent reports of negative emotions as well as greater intensity of negative emotions in individuals with BPD compared to healthy participants [e.g., (9)]. The experimental frustration task increased the intensity of all negative emotions in both groups, except for nervousness, which was higher at the beginning of the study possibly due to the unfamiliarity with the experimental situation. Participants in both groups reported feeling angrier, more frustrated, upset, and embarrassed, which speaks for the validity of the task that is designed to induce frustration and distress. Interestingly, only for anger, we found a significantly stronger frustration-induced increase in women with BPD compared to healthy women. On average, the increase in anger in BPD was more than twice as high as in healthy controls and the group difference was specific for anger. This specificity substantiates results of previous studies. These include experimental studies which, for instance, indicate specific anger biases, like tendencies to misclassify pictures of emotional or neutral faces as angry or recognize anger more often in ambivalent facial expressions [e.g., (35, 36)], but also self-report studies. Individuals with BPD were found to experience anger more frequently, more intensively, and longer, while also showing more anger rumination than healthy controls as well as clinical control groups (10–14). Together these data suggest a specific relevance of anger in individuals with BPD – above and beyond a general negative emotionality. According to the current data, anger might be of particular importance in BPD, as already minor, non-social frustrations lead to significantly stronger increases in anger.

Since anger is closely associated with reactive aggression (18), a frequent interpersonal dysfunction of BPD (5), we were interested whether anger increase was greater in those women with BPD who more frequently act out aggressively in their everyday life and greater deficits in emotion regulation. Using hierarchical linear regressions, we found that aggressive behavior and the level of experienced frustration accounted for 32% of variance in anger increase, while controlling for possible confounding variables such as intelligence, age, and ADHD-symptoms. This underlines the tight associations between frustration, anger, and aggression in BPD. However, contrary to our expectations and previous findings, emotion dysregulation was no significant predictor in the present study. Besides power limitations due to the restricted sample and correlations between the predictors, this non-significant finding remains unclear. A possible explanation could be that the ability to regulate upcoming negative emotions does not play an essential role when asked about feelings of anger immediately after experiencing frustration. Maybe dysregulation makes individuals with BPD more vulnerable to aggression, but does not actually change their frustration-related anger (37). Frustrating situations like the one in the present study may activate maladaptive schemas, such as being attacked, hurt, abandoned, or humiliated by others (38) leading to strong emotional responses—in particular, anger—and eventually aggression as a maladaptive coping strategy (39). This could explain the presents of a specific anger-proneness without overall emotion dysregulation and point to reactivity connected to self-other schemata. It would thus be of interest to know about the goals the participants thought have been frustrated by the task and that elicited anger. Clinical reports and empirical studies suggest that even minor triggers may activate maladaptive schemata of humiliation, defeat, or rejection and the current frustrating situation might be such a trigger (40). Alternatively, individuals with BPD might experience failure in their social rank motive (39) or experiences failure in the task as an intolerable constraint. Beyond a post-experimental exploration of frustrated goals, more assessments of negative emotions would be necessary to measure the duration of high intensity or the time it takes to return to baseline after experiencing frustration.

In a proof-of-concept study, we recently tested a specific group treatment for reducing anger and aggression in BPD (41). This Mechanism-based Anti-Aggression Psychotherapy (MAAP) includes, among others, the identification of contextual factors that enhance the risk for aggression as well as the discussion of cognitive, emotional, physical, and behavioral correlates of anger. Emotion regulation techniques from Dialectic Behavioral Therapy (42) are introduced and practiced to allow participants to monitor their emotions, increase awareness of triggers that initiate anger, differentiate current situations from previously learnt schemata, and dampen anger. Beyond emotion regulation skills, frustration proneness and frustration-induced anger and aggression may become further targets in future treatments that are tailored to reduce aggressive behavior in BPD. The urgency for specific interventions is underlined by the large and significant increase in anger caused by a non-social, experimental frustration task, the prolonged duration (12), and the tendency to ruminate on anger (13, 14), which enhance the risk for aggressive outbursts.

It needs to be mentioned that we did not find any group differences in frustration tolerance or in any other measures of task performance or experienced frustration. On the one hand, this might be regarded as a strength of the current study, since it suggests an equal level of involvement and motivation of the two groups. On the other hand, the data contradict previous reports of reduced frustration tolerance in the TMTT of individuals with BPD compared to healthy controls (20, 21). One reason for this could be the careful matching for age and intelligence and the exclusion of any regular medication in the current study, as well as the relatively high engagement and motivation of all participants taking part in studies of the Clinical Research Unit KFO-256.

Several limitations need to be considered when interpreting the current results: First, our sample only comprised female participants and whether they may be generalized to males remains unclear. Although aggression is generally more frequent in men than in women, sex differences are found to be smaller within BPD samples (43). However, results from neuroimaging studies suggest at least some sex differences in brain correlates of frustration-related anger and aggression (44). Second, we did not include a clinical control group and the specificity of the current results for BPD remain unanswered. However, the specific role of anger is consistent to recent findings from a large ecological momentary assessment study, with different clinical groups including BPD (10). This suggests that enhanced anger might be specific to BPD and should therefore receive specific attention in the treatment of BPD. Nevertheless, further studies including clinical control groups are necessary to further elucidate BPD-specific anger-inducing situations and responses. Third, regular psychotropic medication and substance related disorders were exclusion criteria in the current study to exclude effects associated with these factors which is a strength, but also a limitation of the current study since medication and substance use are frequent among individuals with BPD and the current exclusion criteria might reduce the generalizability of the current results. Fourth, although the sample size was larger than in many other experimental studies, larger samples with more assessments are needed that allow the inclusion of further factors, such as impulsivity, and a more detailed study of emotion dysregulation.

Taken together, the current study confirmed the negative emotionality in women with BPD in general, and further revealed a specific frustration-induced increase in anger, which was significantly stronger in those women who reported more frequent aggressive behavior in their daily life and experienced more frustration during the experiment. In light of this study, future research should consider different sources of anger and aggression in BPD and further investigate frustration-induced increases in anger in social contexts, as well as real-life interactions and monitoring. Anger seems to be a particularly important and prominent emotion for individuals with BPD that needs to be addressed with specific interventions in order to reduce the patient's tendency to act out aggressively, as this is one of the most important interpersonal dysfunctions of this disorder.

## Data Availability Statement

The raw data supporting the conclusions of this article will be made available by the authors, without undue reservation.

## Ethics Statement

The studies involving human participants were reviewed and approved by Ethics Committee of the Medical Faculty of the University of Heidelberg, Germany. The patients/participants provided their written informed consent to participate in this study.

## Author Contributions

KB and SH have received funding for the study, designed the study, supervised data collection, analyzed the data, and written the manuscript. SB and AF have supported data analyses and manuscript writing. CN, MK, KS, and AP have recruited participants and collected data. SH has received funding for the study, designed the study, and supervised data collection. All authors have read, reviewed, and approved the final version of the manuscript.

## Conflict of Interest

The authors declare that the research was conducted in the absence of any commercial or financial relationships that could be construed as a potential conflict of interest.
